# Supplementary material for: Gene-Temperature Interactions and Risk of Childhood Acute Lymphoblastic Leukemia
Source: medRxiv. 2026 Jul 10:2026.07.09.26357608. Preprint. [Version 1] doi: 10.64898/2026.07.09.26357608 (PMC13370497; doi:10.64898/2026.07.09.26357608)
Supplement: Supplementary file 1 [file media-1.pdf]

SUPPLEMENT TO:

Gene-Temperature Interactions and Risk of Childhood Acute Lymphoblastic Leukemia

Tormod Rogne, MD PhD[1,2], Rong Wang, PhD[2], Pin Wang, PhD[3], Kai Chen, PhD[4], Shuangge Ma, PhD[5], Joshua Warren PhD[5], Catherine Metayer, MD PhD[6], Joseph L. Wiemels, PhD[7], Andrew T. DeWan, PhD[2], Xiaomei Ma, PhD[2]

Affiliations:

1) Department of Community Medicine and Global Health, University of Oslo, Oslo, Norway
2) Department of Chronic Disease Epidemiology, Yale School of Public Health, Yale University, New Haven, CT, USA
3) Department of Global, Environmental, and Occupational Health, School of Public Health, University of Maryland, College Park, MD, USA
4) Department of Environmental Health Sciences, Yale School of Public Health, Yale University, New Haven, CT, USA
5) Department of Biostatistics, Yale School of Public Health, Yale University, New Haven, CT, USA
6) Division of Epidemiology, School of Public Health, University of California, Berkeley, CA, USA
7) Center for Genetic Epidemiology, Department of Population and Public Health Sciences, University of Southern California Keck School of Medicine, Los Angeles, CA, USA

Corresponding author:

Tormod Rogne
Department of Community Medicine and Global Health,
University of Oslo,
Kirkeveien 166,
0450 Oslo, Norway
Email: tormod.rogne@medisin.uio.no
ORCiD: https://orcid.org/0000-0002-9581-7384

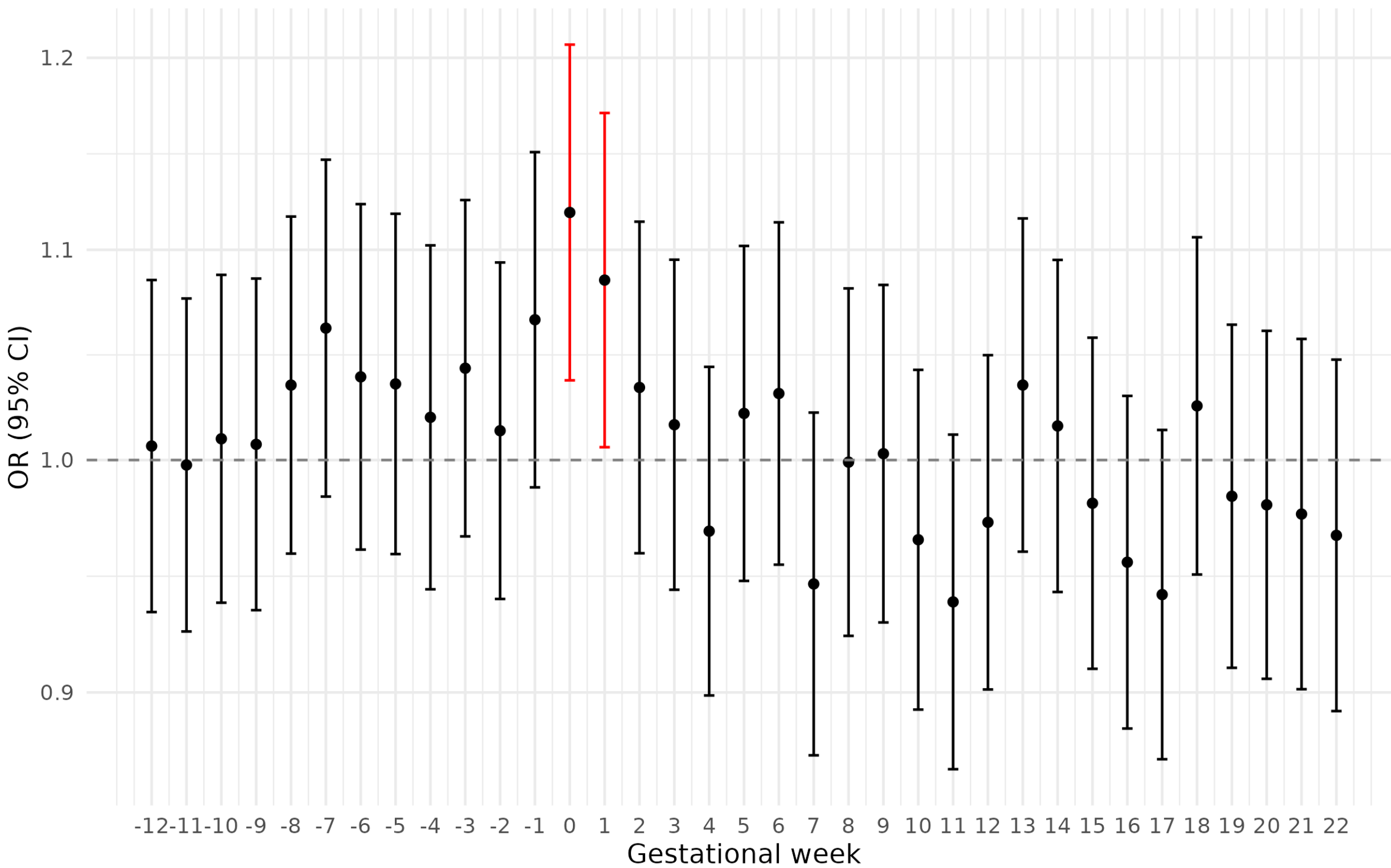

Supplementary Figure 1. Gestational week-specific associations between ambient temperature and risk of childhood acute lymphoblastic leukemia.

Legend: Results from gestational week-specific analyses of the association between ambient temperature and risk of childhood acute lymphoblastic leukemia. Each gestational week was analyzed separately, adjusting for sex, birth year, season of conception, maternal age, birth order, maternal education, neighborhood poverty, county of residence, and principal components 1 to 10. Unit of exposure per 5 °C increase in mean weekly ambient temperature. Statistically significant associations are shown in red. CI, confidence interval; OR, odds ratio.
